# Single-nucleotide polymorphisms and haplotype of *CYP2E1 *gene associated with systemic lupus erythematosus in Chinese population

**DOI:** 10.1186/ar3232

**Published:** 2011-01-31

**Authors:** Ling-hong Liao, Hao Zhang, Man-Po Lai, Shun-Le Chen, Madeline Wu, Nan Shen

**Affiliations:** 1 The Research Base of TCM Syndrom, Fujian University of Traditional Chinese Medicine, Huatuo Road No.1, Fuzhou, 350108, PR China; 2 Department of Biology and Center for Cancer Research, Hong Kong University of Science and Technology, Clear Water Bay Road No.1, Hong Kong, PR China; 3 Joint Molecular Rheumatology Laboratory of Institute of Health Sciences and Shanghai Ren Ji Hospital, Shanghai Institutes for Biological Sciences, Chinese Academy of Sciences and Shanghai JiaoTong University School of Medicine, 145 Shan Dong Middle Road, Shanghai, 200001, PR China

## Abstract

**Introduction:**

Cytochrome P-450 2E1 (CYP2E1) is an important member of the CYP superfamily, which is involved in the metabolism and activation of many low molecular weight toxic compounds. We tried to investigate the possible association of *CYP2E1 *tag single nucleotide polymorphisms (SNPs) with susceptibility to systemic lupus erythematosus (SLE) in a Chinese Han population.

**Methods:**

The coding and flanking regions of the *CYP2E1 *gene were scanned for polymorphisms and tag SNPs were selected. A two-stage case-control study was performed to genotype a total of 876 SLE patients and 680 geographically matched healthy controls (265 cases and 288 controls in stage I and 611 cases and 392 controls in stage II). SLE associations of alleles, genotypes and haplotypes were tested by age and sex adjusted logistic regression. The gene transcription quantitation was carried out for peripheral blood mononuclear cell (PBMC) samples from 120 healthy controls.

**Results:**

Tag SNP rs2480256 was found significantly associated with SLE in both stages of the study. The "A" allele was associated with slightly higher risk (odds ratio (OR) = 1.165, 95% confidence interval (CI) 1.073 to 1.265, *P *= 2.75E-4) and "A/A" genotype carriers were with even higher SLE risk (OR = 1.464 95% CI 1.259 to 1.702, *P *= 7.48E-7). When combined with another tag SNP rs8192772, we identified haplotype "rs8192772-rs2480256/TA" over presented in SLE patients (OR 1.407, 95% CI 1.182 to 1.675, *P *= 0.0001) and haplotype "TG" over presented in the controls (OR 0.771, 95% CI 0.667 to 0.890, *P *= 0.0004). The gene transcription quantitation analysis further proved the dominant effect of rs2480256 as the "A/A" genotype showed highest transcription.

**Conclusions:**

Our results suggest the involvement of *CYP2E1 *as a susceptibility gene for SLE in the Chinese population.

## Introduction

Systemic lupus erythematosus (SLE (OMIM 152700)) is a chronic autoimmune disease with heterogeneous clinical features ranging from mild forms to progressive end organ damage. It is characterized by humoral and cellular immunity to self antigens. Approximately 90% of SLE patients are female. Epidemiological studies suggest the contribution of multiple factors including genetics, environmental exposure and gene-environmental interactions in the etiology of SLE [1-4] The significant difference of SLE prevalence between African descents in industrialized countries and residents in West African countries [5] suggested the combined effects of genetic basis and environmental effect(s) for the clinical manifestation of SLE. Potential environmental triggers for SLE include radiation and exposure to a wide range of exogenous and endogenous xenobiotic compounds [6,7]. Exposure to trichloroethylene (TCE), a volatile organic solvent, was found in several epidemiological studies to induce SLE and some other autoimmune diseases such as scleroderma [8,9]. Autoimmune effects including activation of CD4+ T-cells and an increase of autoantibodies were detected in MRL+/+ mice subjected to TCE exposure [10-12]. Recently, silica exposure from a variety of industrial occupations has been associated with an increased risk of SLE [13].

The cytochrome P-450 enzymes (CYPs) are responsible for the oxidative metabolism and biotransformation of a variety of substrates. The marked inter-individual variability in the expression of CYP genes contributes to the difference in the disposition of many endo- and xeno-biotics, including the metabolites of steroids, environmental toxins and therapeutics [14]. CYP2E1, an important member of CYP superfamily, is involved in the metabolism and activation of many low molecular weight toxic compounds, including ethanol, benzene, nitrosamine and TCE [15]. In MRL +/+ mice, inhibition of CYP2E1 reversed immune-related effects induced by TCE [16]. We undertook this study to evaluate any association between *CYP2E1 *genetic polymorphisms and SLE in a Chinese study population.

## Materials and methods

### Subjects and study strategy

A total of 876 patients with SLE (89% female, mean age 24.9 ± 9.9 years) and 680 healthy individuals (58% female, mean age 51.0 ± 17.6 years) were recruited in the present two-stage study from July 2004 to September 2006 (the first stage) and from October 2006 to August 2007 (the second stage). Unrelated Chinese subjects with SLE according to the American College of Rheumatology (ACR) 1982 revised criteria for the classification of SLE [17] were recruited from multiple medical centers in Zhejiang, Shangdong and Liaoning provinces. All medical records were reviewed to confirm the patient's eligibility and to gather relevant clinical data at the time of diagnosis. Clinical features of the disease were recorded in standardized questionnaires and written informed consent from each participant was also received. The clinical and immunological features of the SLE patients are shown in Supplementary Table S1 in Additional file 1. The controls are area-matched unrelated healthy individuals visiting hospitals. Sample handling and confidentiality protection strictly adhered to the protocols approved by Ethics Committee of The Hong Kong University of Science and Technology. Genomic DNA was extracted from EDTA-treated whole blood using the GFX™ Genomic Blood DNA Purification Kit (Amersham Biosciences, Piscataway, NJ, USA).

In this study, we first screened *CYP2E1 *gene polymorphisms in a panel of 96 healthy individuals. Then the association study for tag SNPs and haplotypes was carried out in a two-stage study approach. During the stage I study, we recruited and genotyped 265 SLE cases (86% female) and 288 controls (81% female) to identify SLE associated SNPs and haploty pes. For the stage II study, we recruited and genotyped 611 SLE patients (90% female) and 392 healthy controls (48% female).

### *CYP2E1 *sequence variations screening and tag SNP selection

For the sequence variation screening the *CYP2E1 *gene region was amplified as eight polymerase chain reaction (PCR) fragments covering a total of 7.5 kb, including the promoter region, exons, exon-intron junctions, as well as the 5' and 3' untranslated regions. Supplementary Table S2 in Additional file 1 shows the primer sequences and PCR conditions used in this study. Direct DNA sequencing was employed to identify the sequence variations. PCRs were performed using hot-start AmpliTaq^® ^Gold DNA polymerase kit (Applied Biosystems, Foster City, CA, USA) and a MJ PTC-200 (Bio-Rad, Hercules, CA, USA) thermal cycler. PCR reaction was carried out in 20 μl solution containing 1.5 mM Mg^2+^, 200 μM dNTP, 0.3 μM each primer, 10 ng genomic DNA as template, and 0.5 U polymerase. Five percent DMSO is included in the PCR reaction mixture, particularly for the amplification of exon 2 and exon 9. After confirming the purity and mobility by agarose gel electrophoresis, each PCR product was purified and subjected to DNA sequencing using BigDye^® ^Terminator v3.1 Cycle Sequencing Kit (Applied Biosystems, Foster City, CA, USA) and the ABI Prism^® ^3100 Genetic Analyzer (Applied Biosystems, Foster City, CA, USA). Each sample was sequenced for both strands to confirm the results and the SNPs were identified by using SeqScape (ABI, Foster City, CA, USA).

Genotyping for tag SNPs rs8192772, rs2070672 and rs2480256 in stage I and II were performed using direct sequencing described above, while tag SNP rs2031920 was detected using previously described Restriction Fragment Length Polymorphism methods [18].

### Statistical analysis

The Hardy-Weinberg equilibrium test was done for each polymorphism detected in the control population. The software HaploView 3.32 (Massachusetts Institute of Technology, Cambridge, MA, USA) [19] was used to calculate linkage disequilibrium (LD) and software PHASE v 2.1 (Department of Statistics, University of Chicago, Chicago, IL, USA) [20] was used for inferring haplotypes as well as analyzing haplotype association. Tag SNPs were selected using Carlson's method [21]. Using this method, bins of the common SNPs that are in very strong LD with a specified r^2 ^threshold were identified and then one SNP was selected to represent the remaining SNPs in each bin. We used an r^2 ^threshold of 0.8 and a minimum minor allele frequency of 0.05. When multiple SNPs were assigned as tag SNPs for a particular bin, the SNP was selected for that bin based on LD pattern, ease of assay design and probability of being functionally important. The alleles and genotypes of the SNPs were counted, and their distributions between the case and control groups were compared by the *χ*^2 ^test to test the hypothesis of association between polymorphism and SLE. Considering the multiple tests involved in this study, a *P*-value < 0.01 was set as significant level. The *P*-value and odd ratios (OR) with 95% confidence intervals (CI) were estimated using conditional logistic regression models adjusted for potential confounders. All the analysis was performed with program SPSS 12.0 (SPSS Inc., Chicago, IL, USA).

### *CYP2E1 *transcription quantification

One hundred and twenty healthy individuals were assessed for the *CYP2E1 *mRNA levels in peripheral blood. The QuantiGene^® ^assay (Affymetrix Inc., Santa Clara, CA, USA) was used to quantify the mRNA considering the accuracy and precision of this technology without RNA purification and RT-PCR [22,23]. According to the manufacture's design and instruction, 10 ul EDTA anti-coagulant blood was lysed at the day of collection. Blood lysates and specific probe sets were incubated in the capture plate and hybridized overnight at 55°C. Pre-amplifier and amplifier reagents were incubated in the capture plate for 60 minutes at 55°C followed by 50°C incubation of label probe with washing between each incubation. Substrate reagents were added in the end and plates were read on a chemiluminescent plate reader. The *CYP2E1 *signal was normalized to an averaged signal from two reference genes *GAPDH *and *ACTB*. All participants were genotyped by sequencing for SNPs rs8192772 and rs2480256. ANOVA test (SPSS 12.0) was used to compare the normalized transcription level among different genotype groups.

## Results

### Sequence variations in the *CYP2E1 *gene

**Figure 1:**
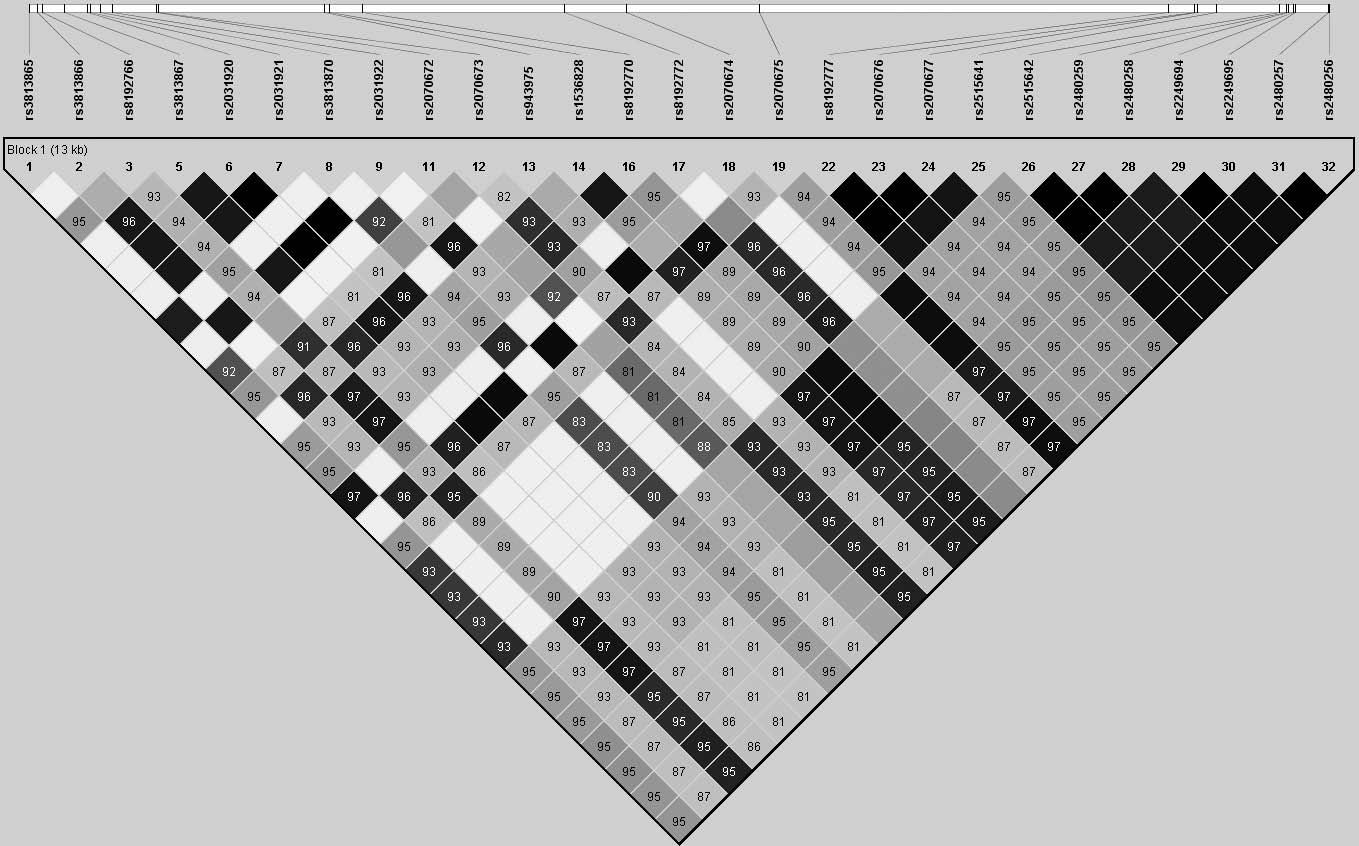
**LD structure of *CYP2E1 *gene region in Chinese population**. LD structure is determined by using the confidence intervals option of Haploview. D prime values are displayed in the squares (values of 1.0 are not shown). Square background colors showed the pairwise r^2 ^value (r^2 ^= 1 for black color and gradually coloring down to white as r^2 ^= 0).

A total of 32 sequence polymorphisms (minor allele frequency MAF >/= 1%) were identified in 96 healthy control samples (12 in the putative promoter region, 2 in the coding region, 16 in the introns and 2 in the 3' UTR). Genotypes were in Hardy-Weinberg equilibrium at each polymorphic locus. Supplementary Table S3 in Additional file 1 and Figure 1 shows the location and frequency of these SNPs. Thirty of them were previously reported in the dbSNP [24] and in the literature [18,25,26], whereas the -1381C/T and -760T/G were newly identified in our study. A high density of SNPs was observed in the putative promoter region, where 12 SNPs were identified within 1.8 kb sequence. Compared with the putative promoter region and the intron regions, the frequency of variations in the coding region is relatively low. Only two synonymous variations, rs28371746 and rs2515641, were detected in exon 6 and exon 8 respectively. We excluded five rare SNPs with MAF less than 5% from LD analysis, haplotype reconstruction and association analysis because rare alleles do not have sufficient statistic power [27,28].

### Tag SNP selection

Pairwise LD across the *CYP2E1 *gene (Figure 1) was relatively strong in this Chinese population and without clear LD block subdivision. All the 27 common SNPs were classified into four bins using Carlson's method [21]. Four tag SNPs, rs2031920, rs2070672, rs8192772 and rs2480256, were selected from each bin for further study based on their allele frequencies, LD pattern and probability of being functionally important. The rs2031920 and rs2070672 were located in the promoter region, rs8192772 was in the intron 2 and rs2480256 was in the 3'UTR.

### Stage I: association of tag SNPs and haplotypes with SLE

In stage I, we recruited 265 SLE patients and 288 controls for genotyping the four tag SNPs. Table 1 showed the distribution of both allele and genotype frequencies of the case and control groups. Association analysis revealed that two tag SNPs were associated with SLE. For tag SNP rs8192772, frequency of "C/C" genotype in cases (5.3%) was almost two-fold higher than in the control (1.7%). Therefore, at genotypic level the homozygous "C/C" genotype was associated with elevated risk of SLE compared with T/T and T/C carriers (OR = 1.912, 95% CI 1.131 to 3.324, *P *= 0.016). In the case of tag SNP rs2480256, association analysis at both allelic and genotypic levels suggested that this SNP contributes to the susceptibility of SLE. The frequency of "A/A" genotype was significantly higher in SLE patients (22.6%) than in the healthy controls (11.8%). The "A/A" genotype carriers have significantly elevated risk of SLE compared with "G/G" and "A/G" carriers (OR = 1.475, 95% CI 1.170 to 1.860, *P *= 0.001). At allelic level, a marginal association was found that the "A" allele carriers showed increased risk to SLE (OR = 1.128, 95% CI 0.999 to 1.274, *P *= 0.053).

**Table 1:** Association analysis of four tag SNPs and haplotypes with SLE in the stage I study

		**No**.					
						
SNP		Controls	Cases	Crude OR (95% CI)	Crude *P*	Adjusted OR (95% CI)	Adjusted *P*
rs2031920							
allelic	C	462	401	1.081 (0.804 to 1.454)	0.604	1.029 (0.887 to 1.195)	0.703
	T	114	107				
geonotypic	C/C+C/T	184 + 94	159 + 83	1.379 (0.585 to 3.247)	0.461	1.151 (0.749 to 0.176)	0.52
	T/T	10	12				
rs2070672							
allelic	A	480	430	0.907 (0.655 to 1.256)	0.557	0.968 (0.821 to 1.142)	0.701
	G	96	78				
geonotypic	A/A+G/G	199 + 7	186 + 10	0.743 (0.504 to 1.096)	0.135	0.753 (0.508 to 1.115)	0.156
	A/G	82	58				
rs8192772							
allelic	T	454	426	0.913 (0.681 to 1.224)	0.542	0.970 (0.837 to 1.125)	0.686
	C	122	102				
geonotypic	T/T + T/C	171 + 112	174 + 76	3.170 (1.126 to 8.925)	**0.022**	1.912 (1.131 to 3.234)	**0.016**
	C/C	5	14				
rs2480256							
allelic	G	360	295	1.282 (1.007 to 1.633)	**0.043**	1.128 (0.999 to 1.274)	0.053
	A	216	223				
geonotypic	G/G+G/A	106 + 148	93 + 109	2.182 (1.377 to 3.459)	**0.001**	1.475 (1.170 to 1.860)	**0.001**
	A/A	34	59				

**Haplotype (rs2031920-rs2070672-rs8192772-rs2480256)**		**Frequency in the Control**	**Frequency in the SLE**	**OR (95% CI)**	***P*-value**		
		
CATG		58.50%	54.70%	0.856 (0.674 to 1.088)	0.205		
**TATA**		**15.70%**	**19.70%**	**1.321 (0.967 to 1.803)**	**0.081**		
CGCA		15.90%	14.50%	0.896 (0.644 to 1.247)	0.513		
Combination of rare haplotypes (frequency <5%)		9.90%	11.10%				

CI, confidence interval; OR, odds ratio; SLE, systemic lupus erythematosis; SNP, single nucleotide polymorphism.

The common haplotypes and their frequency in the patients and controls inferred from PHASE program were listed in the Table 1. Only three haplotypes were presented with frequency greater than 0.05 and all the other minor haplotypes were combined for association study. The frequency of haplotype "TATA" in SLE patients is higher than that in the controls and revealed a marginal association (*P *= 0.081). Since this haplotype "TATA" could be identified from other common haplotypes by genotyping SNPs rs8192772 and rs2480256, we then only genotyped these two haplotype tag SNPs in stage II study with enlarged sample pool.

### Stage II: verifying the association of tag SNPs rs8192772 and rs2480256 with SLE

During the stage II study, we recruited another 611 SLE patients and 392 controls for the genotyping of SNPs rs8192772 and rs2480256. The result confirmed the association tendency revealed in the stage I study (Table 2). For SNP rs8192772, the frequency of "C/C" genotype is higher in the SLE patients than in controls (OR = 1.433, 95% CI 1.023 to 2.009, *P *= 0.037). For SNP rs2480256, both "A" allele (OR = 1.115, 95% CI 1.016 to 1.223, *P *= 0.022) and "A/A" genotype (OR = 1.256, 95% CI 1.062 to 1.485, *P *= 0.008) are associated with SLE.

**Table 2:** Association analyses for rs8192772 and rs2480256 in stage II and the combined two stages

			**No**.					
							
	SNP		Controls	Cases	Crude OR (95% CI)	Crude *P*	Adjusted OR (95% CI)	Adjusted *P*
	rs8192772							
	allelic	T	621	925	1.105 (0.886 to 1.378)	0.375	1.057 (0.945 to 1.182)	0.331
		C	161	265				
	geonotypic	T/T+T/C	242 + 137	365 + 195	1.973 (1.012 to 3.852)	**0.043**	1.433 (1.023 to 2.009)	**0.037**
		C/C	12	35				
**Stage II**								
	rs2480256							
	allelic	G	467	641	1.231(1.025 to 1.480)	**0.026**	1.115 (1.016 to 1.223)	**0.022**
		A	313	529				
	geonotypic	G/G+G/A	140 + 187	191 + 259	1.557 (1.118 to 2.169)	**0.008**	1.256 (1.062 to 1.485)	**0.008**
		A/A	63	135				

	rs8192772							
	allelic	T	1,075	1,348	1.039 (0.873 to 1.237)	0.667	1.045 (0.946 to 1.154)	0.384
		C	283	368				
	geonotypic	T/T+T/C	413 + 249	539 + 271	2.356 (1.344 to 4.129)	**0.002**	1.820 (1.330 to 2.490)	**1.81E-04**
		C/C	17	49				
	
**Stage I + Stage II**								
	
	rs2480256							
	allelic	G	827	936	1.62 (1.092 to 1.460)	**0.002**	1.165 (1.073 to 1.265)	**2.75E-04**
		A	529	756				
	geonotypic	G/G+G/A	246 + 335	284 + 368	1.782 (1.363 to 2.330)	**2.07E-05**	1.464 (1.259 to 1.702)	**7.48E-07**
		A/A	97	194				
	
	**Haplotype rs8192772-rs2480256**		**Frequency in the Control**	**Frequency in the SLE**	**OR (95% CI)**	***P*-value**		
			
	TG		60.36%	54.00%	0.771 (0.668 to 0.890)	**0.0004**		
	TA		18.75%	24.46%	1.407 (1.182 to 1.675)	**0.0001**		
	CA		20.14%	20.05%	0.994 (0.833 to 1.186)	0.944		

CI, confidence interval; OR, odds ratio; SLE, systemic lupus erythematosis; SNP, single nucleotide polymorphism.

When all genotyping data from both stage I and II studies were combined (Table 2), tag SNP rs8192772 was associated with SLE at genotypic level and the "C/C" carrier showed a 1.820-fold increase in SLE risk (95% CI 1.33 to 2.49 *P *= 1.08E-4). For tag SNP rs2480256, it associated with SLE at both allelic and genotypic level. The "A" allele was associated with slightly higher risk (OR = 1.165, 95% CI 1.073 to 1.265, *P *= 2.75E-4) and "A/A" genotype carriers were with even higher SLE risk (OR = 1.464, 95% CI 1.259 to 1.702, *P *= 7.48E-7). Haplotypes comprising tag SNPs rs8192772 and rs2480256 and their frequencies in case and controls were also estimated. Three haplotypes were observed with frequency higher than 5% (Table 2). The frequency of haplotype "TA" was 24.5% in SLE patients, compared with 18.8% in controls (OR = 1.407, 95% CI 1.182 to 1.675, *P *= 0.0001). In contrast, haplotype "TG" is more common in the controls (60.4%) than in the patients (54.0%) (OR = 0.771, 95% CI 0.667 to 0.890, *P *= 0.0004). We also analyzed the association between SNP genotypes and various clinical features adjusted by both sex and age. However, no genotype correlation with any clinical feature was identified.

### Transcription quantification of *CYP2E1 *in PBMC

**Figure 2:**
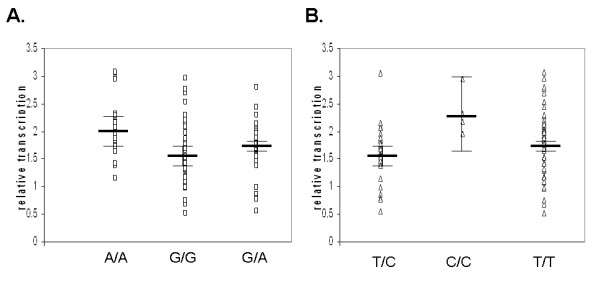
**Relative transcription level of CYP2E1on SNP rs2480256 (A) and rs8192772 (B)**.

In order to study the effect of rs2480256 on *CYP2E1 *gene expression, we further recruited 120 healthy individuals to explore the *CYP2E1 *mRNA level in PBMC. By sequencing genotyping of these 120 individuals, 22 were found as "A/A" genotype, 45 were "G/G" and 53 were heterozygous "G/A". As shown in Figure 2A, the normalized relative mRNA levels from genotype "A/A", "G/G" and "G/A" were 2.01(95% CI 1.79 to 2.23), 1.59 (95% CI 1.43 to 1.76) and 1.74 (95% CI 1.62 to 1.86) respectively. The multiple group test showed the transcription levels among these three groups were significant different (*P-*value = 0.006). The risky genotype "A/A" showed the highest transcription while the lowest is genotype "G/G". We also genotyped SNP rs8192772 and analyzed the transcription difference in different genotypes (Figure 2B). Only "C/C" (2.35, 95% CI 1.68 to 3.03) genotype showed significant higher transcription (*P *< 0.05) comparing to the other two genotypes. The transcription levels between "T/T" (1.78, 95% CI 1.65 to 1.91) and "T/C" (1.60, 95% CI 1.47 to 1.73) genotype did not show statistical significant difference.

## Discussion

After conducting a two-stage study involving the genotyping and clinical evaluation of 876 SLE patients and 680 healthy controls recruited among Han Chinese, we provide the first time evidence for the statistically significant association for SNP rs2480256, of *CYP2E1 *with SLE. The "A/A" carriers for SNP rs2480256 showed higher SLE risk (OR = 1.464 95% CI 1.259 to 1.702, *P *= 7.48E-7). We also detected that haplotype "rs8192772-rs2480256/TG" was overrepresented in controls (OR = 0.771, 95% CI 0.667 to 0.890, *P *= 0.0004) whereas haplotype "TA" was overrepresented in SLE patients (OR = 1.407, 95% CI 1.182 to 1.675, *P *= 0.0001). Though the "C/C" genotype of rs8192772 showed a 1.820-fold higher SLE risk (95% CI 1.33 to 2.49, *P *= 1.08E-4), the haplotype analysis suggested the dominant effect of SNP rs2480256 in SLE susceptibility.

SNP rs2480256 is located in the 3' UTR, a region with proven role(s) in the regulation of gene expression. Many disease-associated variants have been mapped in the 3' UTR of human protein-coding genes [29]. A recent publication on SLE susceptibility exploring also revealed a significant association of a variation located in 3'UTR of *ETS1 *gene [30,31]. The significant association of SNP rs2480256 to SLE maybe due to its effect on *CYP2E1 *gene expression and/or its LD with a functional variant residing in either *CYP2E1 *or neighboring genes. In order to clarify whether this SNP can affect the *CYP2E1 *transcription, we examined the CYP2E1 mRNA level in PBMC sample collected from individuals with different genotype. The statistical analysis showed that *CYP2E1 *mRNA levels are significantly different among different rs2480256 genotypes. The most risky genotype "A/A" showed highest transcription levels, while the protective genotype "G/G" is the lowest. It is also interesting that, for SNP rs8192772, the only risky genotype "C/C" showed significantly higher gene transcription but not "T/C" heterozygous. By examining the LD between these two SNPs, we noticed that only the C allele of rs8192772 is in very strong LD with the A allele of rs2480256 (D' = 1). This explained why the rs8192772 "C/C" genotype showed higher *CYP2E1 *transcription but not in the "C/T" genotype. This transcriptional analysis confirmed the genotyping haplotype analysis proposed dominant effect of rs2480256.

Xenobiotic exposures often trigger the onset of autoimmune diseases including SLE, human CYP genes have significant bearing on individual susceptibility to chemical toxicity and were speculated to be involved in several 'lupus-like' disorders [32]. The inactive CYP2D6*4A allele may be a contributory factor for SLE [33]. After genotyping 90 SLE patients and 94 healthy controls, Yen *et al. *[34] concluded that CYP1A1 4887A may be a precipitating factor for SLE, and they detected a synergistic effect for SLE susceptibility between CYP1A1 4887C/A and Mn SOD 1183T/T. Recent studies in Chinese and Korean further identified Glutathione S-transferase family and CYP1A1 are involved in SLE development [35,36]. As a phase I enzyme, CYP2E1 encodes N,N-dimethylnitrosamino N-demethylase which can catalyze xenobiotics into a more reactive and toxic form. In addition to its ability to metabolize a wide variety of low molecular weight compounds, it is also an effective generator of reactive oxygen species (ROS) [37]. Among the wide substrate spectrum of CYP2E1, TCE has been implicated in the development of autoimmune disorders and immune system dysfunction both in human and animal studies [12,16,38]. TCE is a widely used industrial solvent for degreasing and paint stripping. This volatile organic compound is a common pollutant found in soil and water. Recently, ROS and oxidative stress were implicated in the pathogenesis of SLE [39]. It has been demonstrated that DNA damaged by ROS is highly immunogenic [40,41]. Therefore, it is possible that the higher expression of CYP2E1 contributed to SLE development through the production of more ROS during the compound metabolization with/or particular toxic intermediates produced, for example, metabolites of TCE.

*CYP2E1 *is located in the chromosome 10q24.3-qter, the gene spans over 11 kb and contains 9 exons. When we started this project, the tag SNPs for *CYP2E1 *was not available in the HAPMAP database and literature. We were able to identify four tag SNPs (rs2031920, rs2070672, rs8192772 and rs2480256) by pairwise LD analysis for haplotype inferring. Such detailed information is a good complementary to the released Phase III Hapmap data. And by searching the most updated public database, no other putative or known genes showed significant LD with the SNP rs2480256. In recent years, several genome-wide association studies have been conducted to identify SLE susceptibility markers in different ethnic groups, including the Chinese Han population [31,42-46]. Though validity of these findings has been shown in genes such as *ETS1 *and *SLC15A4*, people also noticed that every study revealed some unique markers. And in these published Genome Wide Association Study data, none of the susceptible markers has a physical linkage with the markers we identified in this study. On the other hand, several genomic scans detecting SLE predisposition genes were performed in extended multicase families or sib-pairs [47-54]. The few overlaps detected among these studies indicate that multiple predisposing genes, ethnic diversity, and genetic heterogeneity are likely. After conducting a 10-cM full genome scan with approximately 400 multiallelic markers for 238 individuals from a multiethnic panel of 62 multiplex SLE families, Cantor *et al. *[47] confirmed the linkage to four previously reported sites (1q23, 2q33, 16q12 to 13 and 17q21 to 23) and revealed four novel sites (3p24, 10q23 to 24, 13q32 and 18q22 to 23). One of these sites, 10q23 to 24, is very close to the CYP2E1 locus. These projects exhibit the complexity of identifying susceptibility genes in a case-control study. The elucidation of disease mechanism and the functional importance of markers involved may eventually guide us to clinical and therapeutic applications.

In this project, we adopted a two-stage association study strategy. In the first stage, all tag SNPs were screened and only the most promising markers were then genotyped in the second stage in other samples. Compared to one-stage designs, two-stage designs can reduce the amount of genotyping and provide near-optimal power to detect the true marker conferring disease risk [55]. In conclusion, the present study suggests that genetic variations in the *CYP2E1 *gene may contribute to SLE susceptibility in the Chinese Han population. One potential concern of the current study is that we only tested limited variants in the functional region of *CYP2E1 *gene and left a relatively large region of introns unexplored, which is also not available in a chip-based genotyping assay. It is possible that, by employing a denser marker map, we may observe some other significantly associated markers which may be involved in regulating gene expression. In addition, replications in other populations and further functional studies are also required to confirm and interpret the association of *CYP2E1 *gene with SLE.

## Conclusions

CYP2E1 gene contributes to the SLE susceptibility in Han Chinese population. The haplotype "rs8192772-rs2480256/TG" was overrepresented in controls (OR 0.771, 95% CI 0.667 to 0.890, *P *= 0.0004), whereas haplotype "TA" was overrepresented in SLE patients (OR = 1.407, 95% CI 1.182 to 1.675, *P *= 0.0001). SNP rs2480256 showed the dominant effect by significantly affecting the *CYP2E1 *transcription.

## Abbreviations

ACR: American College of Rheumatology; CI: confidence interval; CYP2E1: Cytochrome P-450 2E1; LD: linkage disequilibrium; OR: odds ratio; PBMC: peripheral blood mononuclear cell; ROS: reactive oxygen species; SLE: systemic lupus erythematosus; SNP: single nucleotide polymorphism; TCE: trichloroethylene; UTR: untranslated region.

## Competing interests

The authors have no competing interests as defined by Arthritis Research & Therapy, or other interests that might be perceived to influence the results and/or discussion reported in this article.

## Authors' contributions

LHL participated in the sample collection and genotyping, carried out the data analysis and drafted the manuscript. HZ and MPL participated in the sample collection and genotyping. SLC and NS participated in study design and data interpretation. MW participated in the study design and coordination and helped to draft the manuscript. All authors read and approved the final manuscript.

## Supplementary Material

Additional File 1 **Supplementary Tables S1-S3**. Supplementary Table S1: Clinical and immunological features of cases. Supplementary Table S2: Primers for *CYP2E1 *genotyping. Supplementary Table S3: Sequence variations identified in 96 healthy people in *CYP2E1*. Click here for file
