# Supplementary material for: Single-nucleotide polymorphisms and haplotype of CYP2E1 gene associated with systemic lupus erythematosus in Chinese population
Source: Arthritis Res Ther. 2011 Jan 31;13(1):R11. doi: 10.1186/ar3232 (PMC3241355; doi:10.1186/ar3232)
Supplement: Additional File 1 — Supplementary Tables S1-S3. Supplementary Table S1: Clinical and immunological features of cases. Supplementary Table S2: Primers for CYP2E1 genotyping. Supplementary Table S3: Sequence variations identified in 96 healthy people in CYP2E1. [file ar3232-S1.DOC]

Supplementary Table 1. Clinical and immunological features of cases

| Features | **% of patients** |
| --- | --- |
| Malar rash | 45.6 |
| Discoid rash | 4.7 |
| Photosensitivity | 19.8 |
| Serositis | 25.6 |
| Oral ulcers | 21.6 |
| Arthritis | 51.5 |
| Nephritis | 51.3 |
| Autoimmune hemolytic anemia | 15.1 |
| Anti-dsDNA | 72.6 |
| Anti-Ro(SSA) | 59.5 |
| Anti-Smith | 15.3 |

Supplementary Table 2. Primers for *CYP2E1* genotyping

| **Region** | **PCR Primer** | **Sequence (5’-3’)** | **Tm (°C)** | **PCR length (bp)** | **Additional sequencing primer** | **Sequence (5’-3’)** |
| --- | --- | --- | --- | --- | --- | --- |
| Promoter | CP2E1PL | GTTGGTTGGGCATCTAACAGTAT | 61.9 | 1483 | CP2E1PR1 | AGTGAGTCAACCAATTCTGAGAAAG |
|  | CP2E1PR | GCATTTGTTTGGAGTAGGGTTT |  |  | CP2E1PR2 | TTGTGTGTGTGGTTAGAATGAAGA |
|  |  |  |  |  |  |  |
| Exon1 | CP2E1E1L | GGCAGGTGGTGATTCAGGTACTA | 60.0 | 882 |  |  |
|  | CP2E1E1R | CCACAGGCGACACATTTTGAG |  |  |  |  |
|  |  |  |  |  |  |  |
| Exon2 | CP2E1E2L | TCTCTAGAGCAACAGCAATACCC | 56.0 | 929 |  |  |
|  | CP2E1E2R | CAGATAAAGACGAGCCAGACCT |  |  |  |  |
|  |  |  |  |  |  |  |
| Exon3+4 | CP2E1E3L | GAAGAAACTTGCTGTGGAGTCG | 58.4 | 1562 | CP2E1E4L | GGACTCCTAGACTGCATCTGAACC |
|  | CP2E1E4R | TTTTTCTGGATGCAACATAATTGT |  |  | CP2E1E3R | TGTCTGAACCTGTTTGAGTTTCC |
|  |  |  |  |  |  |  |
| Exon5 | CP2E1E5L | CAGGTTTGGAGAAAGTTGTTGGTC | 63.5 | 431 |  |  |
|  | CP2E1E5R | TGACTTGCGTCCAGTGGTCAT |  |  |  |  |
|  |  |  |  |  |  |  |
| Exon6 | CP2E1E6L | CCCTCAGCAGATTTCTGGGAGC | 63.5 | 720 |  |  |
|  | CP2E1E6R | GGGTGTCTTAGTGGGGTATGGAC |  |  |  |  |
|  |  |  |  |  |  |  |
| Exon7+8 | CP2E1E7L | TGTAGGTGGGCAGATGGATAA | 60.0 | 880 |  |  |
|  | CP2E1E8R | GCCTCTGATCTTTCTCACCTGT |  |  |  |  |
|  |  |  |  |  |  |  |
| Exon9 | CP2E1E9L | CAACCAACTCCATACTTTTCACAC | 57.6 | 768 |  |  |
|  | CP2E1E9R | AAAACCTCTCTGTGAGAATCACTTAA |  |  |  |  |

Supplementary Table 3. Sequence variations identified in 96 healthy people in *CYP2E1* gene locus

| **dbSNP ID** | **Position** | **Region** | **Sequence Variant** | **MAF** |
| --- | --- | --- | --- | --- |
| rs3813865 | -1656 | 5' flanking | CCAAAGCCAA **G/C** GCTTCAATTT | 0.224 |
| rs3813866 | -1566 | 5' flanking | TGGACCCCAA **T/A** GGGTGTCTGT | 0.172 |
| rs8192766 | -1515 | 5' flanking | GACAACAGGGT **T/G** CAGGGGTCTG | 0.396 |
| -1414C/T | -1414 | 5' flanking | CTAACCCACC **C/T** GTGAGCCAGT | 0.026 |
| rs3813867 | -1295 | 5' flanking | TTCAGGAGAG **G/C** TGCAGTGTTA | 0.172 |
| rs2031920 | -1055 | 5' flanking | TATAAAAGTA **C/T** AAAATTGCAA | 0.182 |
| rs2031921 | -1027 | 5' flanking | ATTAAGAACT **T/C** CTATATATTG | 0.182 |
| rs3813870 | -930 | 5' flanking | GACTACCTTC **A/G** TAGAAGGTGG | 0.208 |
| rs2031922 | -807 | 5' flanking | AATCGTCTTC **T/C** AAATTTACCC | 0.182 |
| -793T/G | -793 | 5' flanking | TTTACCCTAA **T/G** GTAAAACAAA | 0.016 |
| rs2070672 | -352 | 5' flanking | CCGTTGTCTA **A/G** CCAGTGCCAA | 0.188 |
| rs2070673 | -333 | 5' flanking | AAAGGGCAGG **A/T** CGGTACCTCA | 0.396 |
| rs943975 | 1360 | intron2 | ATTATAGTAA **C/T** AGCATCCGAA | 0.182 |
| rs1536828 | 1415 | intron2 | CGTTGCCTGC **G/C** GAGCGAGGCG | 0.406 |
| rs8192769 | 1684 | intron2 | TGCTCAGCTG **C/T** AGCTGGTGAC | 0.031 |
| rs8192770 | 1744 | intron2 | TCCTGAGACC **G/A** GGAAGGGGGA | 0.391 |
| rs8192772 | 3811 | intron2 | ATGTTGTCGA **T/C** AGATAGGAAA | 0.224 |
| rs2070674 | 4440 | intron3 | CAGGGACCTA **C/T** GGACAAGGAG | 0.177 |
| rs2070675 | 5796 | intron5 | TGTGGGCATG **C/T** ACTCCCCAAC | 0.391 |
| rs28371745 | 6328 | intron5 | ACAAGCAGCC **C/T** CTTCTCCTCC | 0.016 |
| rs28371746 | 6443 | exon6 | GGACAGAGAC **C/A** ACCAGCACAA | 0.016 |
| rs8192777 | 9971 | intron7 | GGTCACTGAG **G/T** GGAAGGGCTG | 0.208 |
| rs2070676 | 10237 | intron7 | TCCTTCAACT **G/C** GAAATATACT | 0.208 |
| rs2070677 | 10274 | intron7 | TATTCAAAAC **T/A** ACATTCTTCA | 0.208 |
| rs2515641 | 10462 | exon8 | ATGGAAAGTT **T/C** AAGTACAGTG | 0.219 |
| rs2515642 | 11113 | intron8 | TGGGCAGACA **C/T** GGTCTTCCCAA | 0.401 |
| rs2480259 | 11176 | intron8 | TTGTATGAAG **A/G** CTGGTCCCCG | 0.401 |
| rs2480258 | 11200 | intron8 | TAGTCAGTGT **T/C** GCTGGTATCC | 0.401 |
| rs2249694 | 11253 | intron8 | TAGGCAGAGA **A/G** GGGTGAGTCC | 0.38 |
| rs2249695 | 11268 | intron8 | GAGTCCTGCC **T/C** TGTGATGGCC | 0.38 |
| rs2480257 | 11609 | 3' UTR | TTCAAACAAG **T/A** TTTCAAATTG | 0.391 |
| rs2480256 | 11614 | 3' UTR | ACAAGTTTTC **A/G**AATTGTTTGA | 0.391 |

MAF: minor allele frequency
